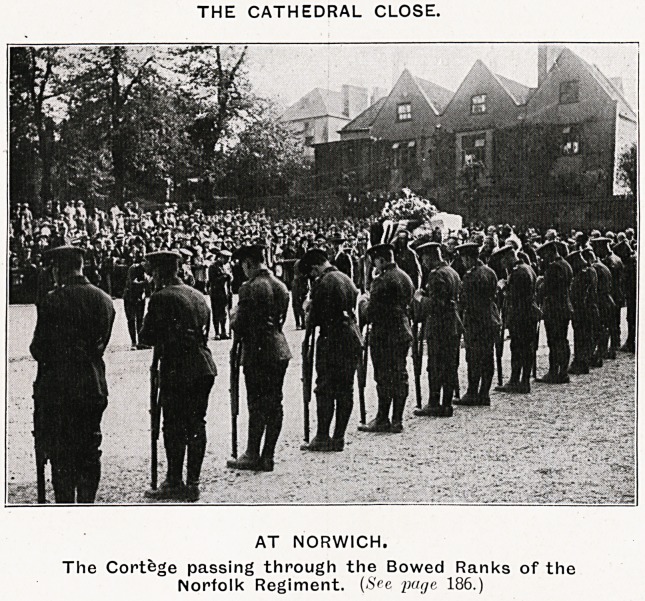# Edith Cavell

**Published:** 1919-05-24

**Authors:** 


### AT ERPINGHAM GATE, NORWICH CATHEDRAL.

The Arrival of the Gun-carriage was witnessed by a Huge Crowd.

### EDITH CAVELL.

The late Edith Cavell was born in 1866. She was the daughter of the late Rev. Frederick Cavell, for forty years Vicar of Swardeston, Norfolk, and of Mrs. Cavell, of College Road, Norwich. In 1895 she entered the London Hospital as a probationer, where she remained for nearly five years, during the latter part of which she was a staff nurse in the Mellish Ward. Then she proceeded to the St. Pancras (North) Infirmary, Dartmouth Park Hill, and won golden opinions from her chiefs. In 1903 she was assistant matron at Shoreditch Infirmary, and proved herself very efficient. She manifested her ability as a teacher, and all the nurses felt the power of her example and the value of the information they acquired through her lectures and classes. Then in 1906 she made her way to Brussels and started the Belgian Training School, of which she wrote at the time: "One of our first duties was to recruit the nurses. The old idea that it is a disgrace for women to work is still held in Belgium, and women of good birth and education still think they lose caste by earning their living. Five pupils have, however, had the courage to come forward, and soon settled in their new life and seem happy in their work." It is interesting to note the excellent method which prevailed throughout the Belgian Training School. Order, and the influence of the matron's personality, were the keynotes which secured the school's marked success. Lectures were given every day in French, the matron being an excellent linguist; there was practical instruction in the wards, and probationers signed on for five years, receiving a salary all the time. The first three years were devoted to training, and the last two to private work or outside nursing. The probationers presented a marked contrast in their neat and hygienic uniform to the nuns in their heavy stuff robes, and the lay nurses in their unhygienic, grimy apparel. The Pope has now sanctioned in some cases hygienic uniform for nuns who are nurses.

Edith Cavell commenced her pioneer work in nursing in Brussels with the desire to spread light and knowledge, which were bound to follow training in the years to come. Her nurses were trained to teach (as none others had at that time the opportunity of doing) the laws of health and the prevention and healing of disease. Belgians who worked under such a matron came to show their countrywomen that education and position do not constitute a bar to an independent life. They proved, to quote her own words in 1908, "a good and solid foundation on which to build a career which demands the best and highest qualities that womanhood can offer." Edith Cavell came over to London and attended the International Council of Nurses in the following year, where it is recorded that the quietly spoken words of the little lady, whose dark hair was already turning grey, impressed all her hearers, who felt instinctively that the future of the Belgian

**EDITH CAVELL**—(*continued*).

nursing profession was safe in her hands. Her staff in Brussels included several fully trained English nurses from the London Hospital and the Shoreditch Infirmary, whose duty it was to attend the private patients in the nursing home attached to the Cavell School, and help in the training of probationers. By 1912 the excellence of the Cavell Home and the training of its nurses were already known and appreciated by the sick and suffering in Brussels.

In 1914, when the war came, Edith Cavell was placed in charge of the Berkendael Medical Institute, while some of her school staff nurses took charge of a board school converted into a hospital when the nurses of German nationality were invited to leave Belgiu . She was a correspondent of the *Nursing Mirror*, and her last contribution was dated March 29, 1915, and contained details of a nurse's life during some of the sad days of the war. Of the Belgian people in the hours of grievous trial she wrote: "I am but a looker-on, after all, for it is not my country whose soil is desecrated and whose sacred places are laid waste. I can only feel the deep and tender pity of the friend within the gates, and observe with sympathy and admiration the high courage and self-control of a people enduring a long and terrible agony. They have grown thin and silent with the fearful strain. They walk about the city shoulder to shoulder with the foe, and never see them or make a sign. Only they leave the cafés which they frequent, and turn their backs to them and live a long way off and apart. A German officer on a tram politely asked a gentleman for a light; he handed him his cigar without a word, and receiving it back threw it in the gutter. Such incidents happen often, and are typical of the conduct of this much-tried nation."

We have here put our readers in a position to understand the noble character, exceptional ability, and high standards of Edith Cavell, with their continuous influence for good upon all with whom she was associated from her earliest days to the commencement of those which preceded the hours of martyrdom and death.

### A Standard for Our Life.

We have spoken of the widespread influence for good which the teaching and example of Edith Cavell had upon all who came under her immediate influence and instruction. The scenes and the attitude of the people through whose ranks her body was borne last week to its final resting-place in the precincts of Norwich Cathedral must bring home to the observants who have a realisation of their spiritual nature and of Christ's life and His teaching that personality in the best of us is undoubtedly an immense power for good by the creation of an atmosphere which reaches out in all directions and touches the souls of others, who may be unknown to and far distant from the possessor of it. Is it not remarkable evidence of the depth of this penetrating and uniting influence that throughout the journeying of all that remained of Edith Cavell men, women and children, sailors, soldiers, and civilians, all classes and types of people, whatever their nationality, spontaneously uncovered and bowed their heads as the gun-carriage passed, whilst the silence was so intense as to be felt by the multitudes who came out to pay tribute to murdered Edith Cavell, whom they had come to accept as the noblest type of woman who, in defence of her own people, bravely faced death and many horrors.

In this connection we hope that the nations of the world, and our own people especially, both at home and throughout the Empire, as well as our American brothers and sisters in the United States, may think out for themselves the well-nigh illimitable influence for good, through human sympathy and tenderness, which is brought to all classes and individuals who have the privilege of paying their reverent acknowledgments to its power and uplifting. It found instant expression in the thousands and thousands of people who lined the routes on land throughout the long journey from Brussels to Norwich. This influence was unconsciously made manifest in many ways. Everywhere the crowds opened and the children carefully made room for, and placed in front. It seems as if there was a deep meaning underlying this action recalling Christ's words: "Unless ye be a little child ye cannot enter the Kingdom of Heaven." Men and women, as we have ventured to express our conviction, had, for the most part at any rate, awakened to the realisation of their spiritual nature, and here were these innocents brought to the front, and treated with tender consideration by the crowds through whom Edith Cavell's remains were borne.

Edith Cavell's influence during life cannot, in God's mercy, perish with her death. The atmosphere which she created, and which her life's work has unconsciously spread through the peoples already, must assuredly quicken the understanding in men and women of the glories of such an influence, and the desire, by personal service and example, with the yielding up of self, to acquire a standard of life which will bring to them the wish to follow her life, and go and do and live like Edith Cavell. Every one of us can find a standard for our life, and those who, by prayer and purposeful endeavour, seek to maintain that standard in all things at all times, unceasingly, can by perseverance obtain, as the experience of the best amongst us proves, **the Peace which passes understanding.** Whatever their fate, wherever their lot may be cast, in all circumstances, at all times, they will then find the troubles of this world, however acute, cease to possess the power to disturb or break the serenity of mind which is ever theirs.

### Patriot and Martyr.

Up to May 15 we candidly confess there was at the back of our mind, as we have since found there has been at the back of a good many people's minds, the question whether the exhumation of Edith Cavell's body, and its transference to its present resting-place were completely justified. The underlying cause of this we believe to have been that the actual circumstances which led up to her

General View of the Procession at Dover.

**EDITH CAVELL, PATRIOT AND MARTYR**—(*continued*).

arrest and execution were not known, save possibly to a very few official people. The *Daily Mail's* correspondent, Warrior Ferdinand Tuohy, learnt the facts at Ypres in 1915, in association with the late Captain E. D. Paine, a former member of the staff of the *Evening Standard*, when they were commissioned jointly to edit the Christmas number of the soldiers' paper called *The Salient*. We are indebted to Warrior Tuohy's article, published in the *Daily Mail* of May 15, for the facts here given, which follow. Warrior Tuohy and Captain Paine in their capacity as joint editors received a MS. entitled "A Modern Scarlet Pimpernel," across which a General Officer had scrawled "Go very carefully with this; we don't want to give anything away." The narrative, it was found, gave the system upon which Edith Cavell had worked, in conjunction with a Prince de Croy or Crouy, and Warrior Tuohy adds it had to be mercilessly blue-pencilled before it could appear, even in *The Salient* with its circulation of eighty copies. At this time Miss Cavell had only been buried a few weeks, and the publication of the slightest additional information might have materially helped the Germans through von Bissing in his ruthless methods in Brussels. The Prince de Croy, a Belgian nobleman, and his sister, the Princess, devoted themselves to the task of getting Allied soldiers out of Belgium into Holland during the first months of the war. The Prince's château was in the district of Mons. The big German advance "swept up and over it like a tidal wave," leaving behind it a scattered jetsam of detached officers and men, the collection and returning of whom to England now became a self-imposed task of the Prince and his sister. In fulfilment of her task the Princess was in the habit of going to Brussels in a cart, disguised as a peasant woman, to see an old friend, Edith Cavell. Miss Cavell thus learnt that the château at Mons was to be the rallying-centre for all Allied fugitives, both wounded and unwounded. The peasants were to lead the fugitives there by night, and the signal was to be sand thrown at a certain window. Forwarding agents along the line of route to the Dutch frontier would give food and shelter to the fugitives, when they arrived attended by duly accredited guides. The Princess asked Edith Cavell to act as one of three such forwarding agents in Brussels. This she readily agreed to do, having already been sheltering English fugitives of her own accord. Her task was to keep the men in Brussels till they could safely be got away to the frontier. When told that the Germans had threatened to shoot anyone caught harbouring Allied soldiers, Edith Cavell's reply was, "We must take the risk. We are doing no harm, only helping our own people. A German woman would do the same." The Princess gave directions that excessive caution was necessary, and warned her to have nothing to do with men not accompanied by one of the guides, whose code-word was the day of the week, and who were all under Bourg.

As time passed the Germans got much stricter, and only parties of two or three could be got through to Brussels, and they had to have false identification papers prepared. The Princess photographed these men, while the Prince forged the signatures and stamps. Edith Cavell herself took the men to the rendezvous. To prevent suspicion her plan was to send her fugitives, being French or Belgian, into the crowded streets by day, and if English to give them work as orderlies in her hospital. Thus everything, when the Germans called, was in order. Fugitives who were caught in civilian clothes or uniform were shot out of hand. The smuggling of fugitives into Holland proceeded through the spring and summer of 1915. "Miss Cavell was splendid. She went on with her own work all the time, nursing Germans, French, and Belgians. She never made a slip from beginning to end. She was ultimately given away by one or two of the men she had saved writing to thank her. The Germans intercepted these letters."

Attempts were made by the Germans to entrap Edith Cavell by sending to her house two agents who posed as fugitives from the Mons Château. Unaccompanied as they were by a recognised guide, she simply said she could not take them in. Then Bourg was arrested, and Edith Cavell warned the Prince, who disappeared, but he would not have gone had he known of the wholesale arrests to follow. A week later thirty persons, including the Princess and Edith Cavell, were arrested. Few were allowed a lawyer, and to entrap the prisoners when they kept silent, agents masqueraded as fellow prisoners in the cells. The bogus prisoners succeeded with some of the prisoners, but not with Edith Cavell. There was no real evidence against her, but she refused to deny having helped our soldiers. To the end she thought of others, and when sitting beside the Princess at the trial pretended not to know her. At 9 P.M., on the night fixed for Edith Cavell's execution, neutral diplomats hurried to the Theatre Royal, where von Bissing and von der Lancken were enjoying themselves. "The matter is entirely in the hands of the military governor," said von Bissing. "Monsieur," said the Spanish Minister, "you are committing worse than a crime; you are committing an error."

**It is declared that the hand that shot a great heroine is the hand that kept Rantzau sitting at Versailles. No change. Warrior Tuohy states it may be well if all our own people, and those of our Allies throughout the world, when they think of Edith Cavell should remember this, and especially the British Army on the Rhine surrounded as our men are by every form of reptile servility.**

The more widely this authentic narrative is known of Edith Cavell's work for her countrymen during the weeks preceding her execution, together with her life's history and character, the more gratefully will the people of the nation and the Empire welcome her exhumation and re-burying at Norwich, the home of her family, within the precincts of its ancient cathedral. The universal testimony of admiration for the heroic and noble conduct of her inspiring life, which has been exhibited with impressive reverence by all classes in the countries

**EDITH CAVELL, PATRIOT AND MARTYR**—(*continued*).

through which she passed, should make a lasting impression on mankind, and lead to the extension, if possible, of her influence for good, and the uplifting of the peoples throughout the nations.

Buried by her murderers in obscurity, so secret was her sentence, and so shamefully hurried its execution, we might never have known at the time how great an Englishwoman Edith Cavell was, as the *Times* points out, but for the efforts of the American Minister to save her life. "Of the charge brought against her, of assisting English and Belgian soldiers to escape, she was nobly guilty. Her trial was by our English ideas unfair, but no ingenuity of defence could have obtained acquittal. She knew all the risks she was taking in helping hunted fugitives to escape from almost certain death, and there was more courage in the sustained struggle of her woman's wits with the enemy, than in the hectic flush of the average battlefield. Such virtue would have shone bright anywhere; but surely no enemy but the German would have been so dully malign as to give it the foil of her execution. To this day the Germans never seem to have understood quite why her execution made such a stir in the world. Germans cannot see how much greater the sympathetic beating of a warm heart is than the grinding of any machine, even so big a machine as the German army, and how much more heroic love is than hate. Edith Cavell did what she did partly because she was English, and there were Englishmen to be saved, but perhaps even more because she was a woman, and her womanly heart went out to man's helplessness. . . She hated no one, and in her hospital she nursed German soldiers as well as English and Belgian. There she was a true nurse, and our care for her profession, that has cared so much for us, will be the best monument to her memory."

### ALL THE NATIONS BOW THEIR HEADS.

At Brussels, at Dover, in London, and at Norwich the heartfelt tribute to the memory of Edith Cavell was identical in its depth of feeling. All the nations, all classes of people bowed their heads in reverence and sympathy. At Brussels it is declared that that city has never witnessed a more impressive spectacle than that of the transfer of Nurse Cavell's body from the Tir National to the Gare du Nord. Every house flew a flag at half-mast; the streets were lined with crowds; the organisation was excellent in its simplicity. The *Times* states:—"Of the procession and the religious service at the station it may be confidently affirmed that no Englishwoman except Queen Victoria ever had a more moving or a grander progress to her last resting-place." At the station the Central Hall had been transformed into a mortuary chapel. The coffin was reverently borne into the station, and the Rev. H. S. T. Gahan, the British Chaplain, read the opening sentences of the burial service. He then read portions of 1 Corinthians xv. in French and the remaining prayers in English. Afterwards, as the coffin disappeared into the funeral car at the end of the train, the band played "God save the King." The silence, so deep and unfamiliar in these surroundings, was awe-inspiring. No sound suggestive of the ordinary life of the station was to be heard. Behind cordons of Belgian troops stood nurses of the Red Cross and members of other women's corps attached to the armies, and behind these were several hundred invited spectators.

AT DOVER.
Sailors carrying Wreaths in the Procession.

At Ostend the body was removed by General Ryckel, Acting Burgomaster Moreau, and the British naval and military authorities. Honours were paid by a detachment of Chasseurs, and a wreath was laid on the coffin in the name of the town of Ostend. The coffin remained throughout the night in the mortuary chapel under a guard of the Belgian army. It was brought home to England from Ostend by the destroyer *Rowena* (Lieut. Commander Lawrence D'O. Bignell), escorted by the destroyer *Rigorous*. As they approached Dover Harbour the ensigns of the ships of war within it were lowered to half-mast. In due course six bluejackets bore the coffin to the wheeled bier that awaited it. It was covered with the Red Cross flag. Preceding it went a party of bluejackets carrying the wreaths, which included one of palms and evergreens bound with the national colours from the King and Queen of the Belgians, another from the hospitals of Brussels, a third from the French and Belgian political ex-prisoners, "who would have been her companions in captivity

ARRIVAL—IN LONDON.
Procession leaving Victoria Station.

THE HOUSES OF PARLIAMENT AND WESTMINSTER ABBEY.

After the Service: Leaving Westminster Abbey.

**EDITH CAVELL: ALL THE NATIONS BOW THEIR HEADS**—(*continued*).

if Miss Cavell had not been barbarously shot by the Germans." A lovely wreath of pink roses from the Mayor and Corporation of Dover, with the card "In remembrance of a British heroine."

The pier had been kept clear of the public, but when the cortège reached the pier gates, where the military aspect grew more imposing, it was the people's tribute which found expression. The Marine Parade was densely crowded, and throughout the entire route huge numbers stood awaiting the procession to pass. Sailors and soldiers figured largely in the throng, and came briskly to the salute as the coffin appeared. Everywhere there was the deepest reverence, and the thousands of little children who lined the route seemed to know that they were witnessing the nation's homage to one whose name will ever figure among the Empire's illustrious dead. Under a military guard the coffin was placed in an open hearse, and was accompanied by sixteen pall-bearers, four of them officers of the Women's Royal Air Force, four officers of Queen Mary's Army Auxiliary Corps, four officers of the Women's Royal Naval Service, and four Army nurses. It proceeded to the Marine Station, where it was placed in a specially prepared *chapelle ardente*, with a double guard of sentries commanded by Lieut. O'Leary, V.C.

Through the Home Land.

On Thursday the coach containing the coffin, together with a special coach for the mourners, left Dover at 7.30 a.m. Few of the nation's heroes who have travelled through Kent towards the capital, for welcome or for burial, can have stirred public feeling so deeply, and none in quite the same way as it was stirred on Thursday, the 15th instant, by the last journey of Edith Cavell, and rarely have funeral scenes had such a background. The orchards of Kent were in full blossom, the fields golden with buttercups and every bank blue and white with wild flowers. England had put on beauty to receive back her own. At almost every station along the line, at windows near the railway and by the bridges, there were crowds of children quietly and reverently watching the passing. Schoolboys and schoolgirls in bright summer clothes had been brought by their teachers to the railside, and they stood in long lines three and four deep on the platforms. The boys saluted and the girls stood at attention. At Sittingbourne quite 2,000 people were assembled.

London's Sympathy and Admiration.

It has been truly said that Edith Cavell's funeral, entirely spontaneous in character, as the numbers who attended it throughout testified, was without precedent. The *Times* records that as the solemn procession came out of Victoria Station into the wide avenue of Victoria Street thousands of men bared their heads, soldiers—including Canadians, Australians, New Zealanders—stiffened to attention, officers saluted, women and children stood in reverent quiet. There was no motion in the multitude, and no sound came from them. But for the roll of the drums, the beautiful melody of the Funeral March, and the slow, stately tread of the escorting Guards, the silence would have been unbroken. Overhead there was a cloudless sky. Sunshine lit up the crowd and made a golden way for the passage of the gun-carriage and its honoured burden. From the buildings scores of flags flew at half-mast. Among them could be observed the colours of the Dominions of South Africa, Australia, and Canada, and a solitary White Ensign drooping from a fifth-floor window. Along the route to the Abbey there was no break in the line of mourners. Mourners the people can be called, for while it is probable that no one man or woman in the crowd ever saw Edith Cavell, they had gathered to express a deep sympathy born of admiration for what a woman, surrounded by her country's enemies, did for England. So it was, with the flag above the gun-carriage covering the honoured dead, and the silence everywhere deepening, that, preceded by the Guards, the coffin was brought round to the great entrance to Westminster Abbey. Likewise everywhere throughout the route traversed after the service, along the Embankment, through the City to Liverpool Street station, similar scenes were enacted, and equal reverence and whole-hearted sympathy and devotion were manifested. London has probably never experienced anything like it in its long history. During the passing of Edith Cavell through London a wonderful silence rested over streets which at the midday hour are usually clamorous with sound. The tribute offered by a mighty crowd to the dust of a very gallant woman was a tribute of silence, but in that silence the dead was acclaimed as surely and splendidly as the living heroes of the War have been welcomed home by the cheers of the people.

### In Westminster Abbey.

### Mors Janua Vitæ.—May 15, 1919.

There could not be more perfect English May weather than that in which all that was mortal of Edith Cavell came back to England, and was received on the way through London to the last resting-place at Norwich, not so much with lamentation as with a joyful welcome. London streets were thronged with people, and as the procession drew near to Westminster Abbey the first sound heard by the crowd within was that poignant melody of superhuman happiness from Chopin's Funeral March, the Song of Songs for Christian faith in the presence of death.

Before the high altar six tall red candles burned beside a waiting bier, pale in the golden sunshine that flooded the glorious old church, while the band of the Grenadier Guards filled it with music.

**EDITH CAVELL: IN WESTMINSTER ABBEY**—(*continued*).

Among those who gathered to honour the dead nurse were many distinguished and familiar faces. Besides Queen Alexandra and Princess Victoria, Lord Athlone represented the King. There were representatives of foreign countries, English statesmen, doctors, scientists, representatives of hospitals and institutions, leaders of all manner of good works, deputations from Women's Auxiliary Services, and, of course, many heads of the nursing profession, and many more of the rank and file, who were themselves honoured in the honour offered to Edith Cavell.

There was the same sense of life triumphant over death in the singing to Dr. Croft's music of the Burial Service sentences as men of the Grenadier Guards carried the coffin up the long aisle. The Union Jack draped it, and upon this again lay Queen Alexandra's large cross of scarlet and white flowers. When it was placed on the bier, and the clergy, in their heavily silvered black velvet copes, had passed to their places, the softly chanted twenty-third Psalm carried on the train of thought to rest in green pastures and the picture of one not afraid because not alone in the Valley of the Shadow of Death, not even in that grey dawn in Brussels, three and a-half years ago, in the face of enemies who had no pity when they believed themselves conquerors.

The Lesson went yet further, and gathered together into a great ideal all our aching longings for the bettering of the world, all the feeling out after practical schemes of reconstruction. "I saw a new heaven and a new earth . . . the tabernacle of God is among men. . . . He that sitteth on the throne saith: Behold I make all things new. . . . He that overcometh shall inherit these things." The Revised Version was used.

Of what use, indeed, is the self-sacrifice of those who loved not their lives even unto death, but gave them freely for us, unless we build on the foundation they laid? Edith Cavell broke the laws of oppressors to help the persecuted. She saved others, herself she could not save. She went to her death with the majestic saying that merely to forgive is not enough. Her trial in its high-handed illegality was a mockery, and the whole ceremony in Westminster Abbey was infinitely more than the national honouring of a noble woman cruelly done to death. It was a national protest against the spirit of selfish greed, of heartless rapine, and the lust for domination which has caused an infinite number of violent deaths that were undeserved, and an untold mass of suffering that will linger on through the life of a whole generation. There was Captain Fryatt, the fearless protector of lives entrusted to his care. There were the North Sea fishermen, the *Lusitania* victims, the women and girls of Lille sent to slavery, and how many more? Those things are past, the War is past, the present is ours in which to make a better future. "Show Thy servants Thy work, that their children may see Thy glory."

THE CATHEDRAL CLOSE.

AT NORWICH.

The Cortège passing through the Bowed Ranks of the Norfolk Regiment. (*See page* 186.)

After the Lesson followed prayers, and then the hymn "Abide with me," henceforth to be always associated with the memory of Nurse Cavell. The congregation took it up with evident emotion. Then the Blessing and the Dead March from "Saul." The sounding of the Last Post from the triforium in the choir had a wonderful effect in our great Abbey Church, but more impressive still was the réveillé of drums. Beginning faintly, and as if at great distance, the volume of sound swelled into a noise like that of the greatest of storms or the marching of humanity in one vast army. It died away again into a softness as of infinite distance, and the great congregation passed out after that little coffin and its soldier-bearers into the brilliant day, stirred—let us believe—by more than a temporary emotion. Some followed as mourners to the last resting-place in Life's Green at Norwich of the woman who had played her noble part, the rest went back to the every-day matters of life, sobered perhaps, but assuredly not depressed, encouraged, perhaps, to try to make life a better thing because of a moment of vision which had shown death as the gate of a fuller and truer life beyond.

**EDITH CAVELL**—(*continued*).

### The Ceremony at Norwich.

Thursday at Norwich was a perfect spring day, when the ancient city displayed its characteristics with the maximum charm. The effect of the ceremony at Norwich was one of simplicity. The people were gathered together by the railway station and in the streets in the neighbourhood of the Cathedral in quiet groups. The special train arrived at five o'clock in the evening, with a military escort of the Norfolk Regiment. The coffin was taken on a gun-carriage from the station to the Erpingham Gate (see p. 177), passing the monument to Miss Cavell unveiled by Queen Alexandra last year. The pall-bearers were: Miss Cann, R.R.C., matron of the Norfolk and Norwich Hospital; Miss Arnold, Q.V.J.I., superintendent of the Cavell Home at Norwich for District Nurses; Miss Fowler, Q.V.J.I., county superintendent, Norfolk Nursing Federation; Mrs. Mahon, commandant of the Town Close V.A.D. Hospital; Mrs. Steele, commandant of the 62nd Norfolk V.A.D. Detachment; and Mrs. Hales, quartermaster of the 64th Norfolk V.A.D. Detachment. Nurses walked before the gun-carriage, and private mourners and friends followed it, with representatives of the Anglo-Belgian Union and of the Edith Cavell Homes of Rest.

As the body was carried up the nave of Norwich Cathedral, accompanied by a long procession, it passed through guards of commandants and nurses of the Norfolk Branch of the Red Cross. The coffin was almost covered by one immense cross of red and white carnations sent by Her Majesty Queen Alexandra, and a small bunch of flowers inscribed "To a noble woman from a humble woman." The service included a commendatory prayer giving thanks for a noble example of courage and devotion for the well-being of others. The singing of the Cathedral choristers was very beautiful, the purity of the boys' voices being most marked in a Cathedral which has fewer perplexing echoes than is usual.

The grave was in a green corner under the very shade of the Cathedral, which had been specially consecrated. At the conclusion of the service the Bishop said:—

"Here we welcome the dear form of Edith Cavell among her own people, to leave her where the new chapel is soon to be built in memory of Norfolk sailors and soldiers who gave their lives for us. The tribute of the Empire has already been paid in London, and at this peaceful spot I call your minds away from the distressing military and diplomatic aspects of her last weeks to dwell, not on the work of her life, but the manner of her death.

"My mind goes back to All Saints' Day, 1915, when I visited her aged mother and we said together the beautiful collect for All Saints' Day, that collect so dear and sacred to many whose treasure is in Heaven. The Roman poet speaks with pity of dead boys and unwed maids and the young cut off before their parents' eyes. But from Christ we have something far better than pity. He has brought to us hope eternal, and to-day we think of mother and daughter together once more where, in Newman's beautiful words,

> Now they join hands once more above
> Before the Conqueror's Throne,
> Thus God grants prayers, but in His love
> Makes ways and times His own.

"Edith Cavell rests under the shade of our Cathedral in its 800th year, adding one more to the long line of those blessed saints of God over whom it has watched in life and death. This plot of ground is now called 'Life's Green,' and we will think of her this evening, while her body rests in its keeping, as herself alive unto God and present with the Lord, and we will look on to the glad day when she and we and all we love, having waited and watched, here or there, for the glory of the Resurrection, at last shall see—

> The splendour of the morning
> Down on the Hills."

The last hymn at Norwich, as at Westminster Abbey, was "Abide with Me." Following it was the Blessing, pronounced by the Bishop. The "Last Post" was sounded over the grave, and the "Nunc Dimittis" concluded the ceremony.

### The Passing of Edith Cavell.

We take the following from the *Daily Telegraph* of May 15:

"When Edith Cavell was called to her last account she gave by her death one more shining proof that he who loses his life in a sacred cause gains a larger and fuller life in the world's grateful recognition of a magnificent example. It is not only that her name has become a symbol of all that we cherish in heroic womanhood. There is something more in a case like this than the brave acceptance of a dreadful doom. No man, says the Scripture, liveth to himself, and no man dieth to himself. From a central fact like the death of Edith Cavell various concentric rings of thought and influence radiate through the world, just as a stone thrown into a pond will initiate a series of circling waves along the whole expanse of the water. Germany never did a worse thing for her fame and her future than when she took vengeance on a nurse for an act of charity. Edith Cavell never could have done a greater thing for her country than when she confronted her executioners as a sacrifice to prove to the world at large the innate coarseness and brutality of the Teutonic mind. For henceforth it became matter of common notoriety that in fighting against Germany we were not only struggling against a nation which made war upon defenceless women, but also championing the cause of justice and mercy and the finer graces of human civilisation, all alike threatened by the dark shadow of a merciless tyranny. Thus the name of a devoted nurse became for us at once a challenge and a battle-cry, and her murder a signal for a determined and unceasing war against the race which had ordained her execution. It was but one woman who was butchered. But it was a woman to whom

EDITH CAVELL: THE PASSING OF EDITH CAVELL—(*continued*).

had come the unique opportunity of representing in her single person the gracious influences of pity and tenderness which we associate with our Christian faith and our best ethical ideals. Edith Cavell died a martyr, and like all martyrs gave an inspiring message to the world. 'Be of good comfort, Master Ridley,' said Latimer, when both were burnt in Mary's reign at Oxford, 'for we shall this day light such a candle, by God's grace, as I trust shall never be put out.' To heroes and heroines of this calibre there is no such thing as death, for they illustrate for us anew the beauty which never fades, the eternal beauty of holiness."

### An American's Thoughts.

### German Savagery Unveiled.

We have already pointed out how strenuously the American Minister laboured to save Edith Cavell's life. In this connection the Americans within reach rendered heartfelt, continuous, and loyal service. Edith Cavell's execution stirred our American relatives to the depths, to which an American writer and novelist, Mr. Owen Wister, bore striking testimony in *The Times* of the 16th instant.

"By two deeds that she did in the earlier days of the war, more than by any that she did before or after, Germany hurt herself with America. These were the sinking of a ship, May 7, 1915, and the shooting of a woman, October 12, 1915. If ever there had been a possibility that American sympathy might be so divided as to hold us back from our duty and our salvation, that possibility was killed for ever when Edith Cavell died for England. So it may very confidently be said that, if Germany's doom was not sealed already, she sealed it herself by those two acts in 1915. In the hour of Edith Cavell's funeral service, as her body approached Westminster Abbey, but before it had quite reached the entrance, the waiting people thought it was come and rose in silent respect. During that silence, very faintly from above us as we stood, the clock struck noon.

"As I counted the strokes, these were my American thoughts:—

"This woman, who died for her country, died for more than that. The shots by which she fell killed what was left of the chance we should stay out of the fight. They tore away whatever was left of the veil that hid German savagery from our eyes. After that, it was merely a question of time when our bodies and our spirits should be equipped to join in defeating Germany. This Edith Cavell did; and now to-day, here comes her body, and we all, of many nations, but mostly of British race, rise to meet its approach, united in reverence and gratitude beneath this roof.

"Presently the singing began, and my thoughts went on:—

"These words from the Bible that I am hearing, these prayers, this hymn, 'Abide with me,' are the corner-stone of both my faith and my speech. The prose of the Bible is the foundation upon which rest my belief, my law, and my manner of expressing them. This roof, where these words I have known by heart all my life are being now uttered, is the shrine of my history. It belongs to me. It cannot be possible for any American, however untravelled hitherto, to enter here and linger awhile and learn what it holds, not to be stirred to his depths. The place speaks to him of himself, his meaning, his past, the great race to which he belongs. May the striking of that noon hour and the coming here of Edith Cavell's body mark the end of the era of misunderstanding and the beginning of the era of understanding between Great Britain and America."—Owen Wister.

### A Brave Englishwoman and After.

Edith Cavell was a good woman and a Christian, with an abiding faith. The Bishop of Norwich mentioned that on All Saints' Day, 1915, after Edith Cavell's death, he visited her aged mother, and they said together the beautiful collect for All Saints' Day, that collect so dear and sacred to many whose treasure is in Heaven. We have given a very full and exhaustive account of the wonderful revelation which Edith Cavell's death has called forth in the foreign and English cities through which all that remained of her was carried to its last resting-place. We have ventured already to point out this unique and memorable fact, and the lessons and opportunity it affords to the ministers of religion and Christian people everywhere. Is the inspiration of her life and splendid example to end with her death, or even with the multitudes who gathered in reverence to render tribute to her life and character? Is the work of compassion which she began in Brussels to have no effect upon our own individual lives, and especially upon the individual life of her sister nurses of all ranks, whom she leaves behind? We cannot believe any such grievous fate awaits us. We share the belief which Edith Cavell, there is reason to know, had in her heart, that the work of compassion which she began would survive her. Only two days before the German bullets pierced her breast she wrote to the nurses of the Belgian Training School at Brussels: **"When brighter days come our work will resume its growth and its power for doing good."** And a little later she wrote again: **"I have told you . . . that devotion will give you real happiness; and the thought that you have done before God and yourselves your whole duty and with good heart will be your greatest support in the hard moments of life and in face of death."**

The Editor hopes, and will ever pray, that all who have the privilege of studying the life and lessons of Edith Cavell may resolve with prayerful determination that henceforward each one of them, if they do not already possess it, will set themselves a standard for their life. Then, in Edith Cavell's words, devotion will give to every one of us real companions. Then, if we stand by our standards, each one of us, in Edith Cavell's words, will find with this good woman that devotion will give to each of us real happiness throughout life and in the face of death.

## Figures and Tables

**Figure f1:**
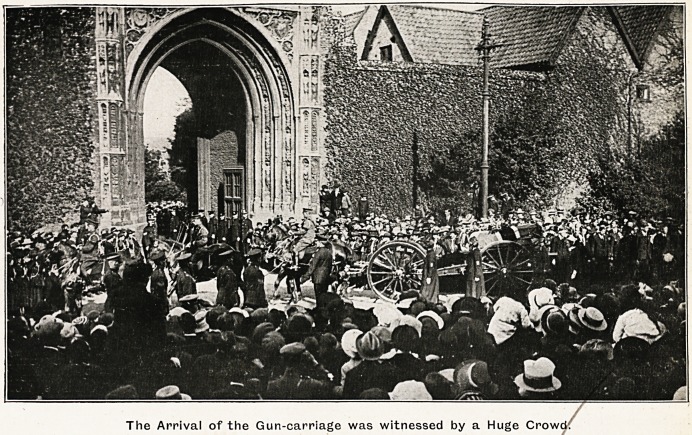


**Figure f2:**
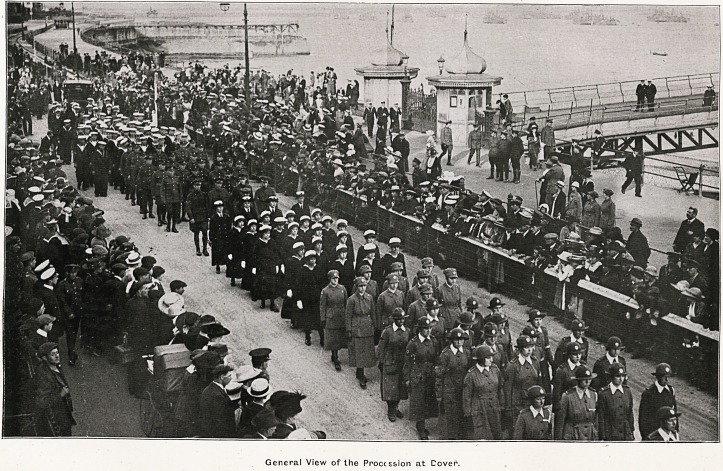


**Figure f3:**
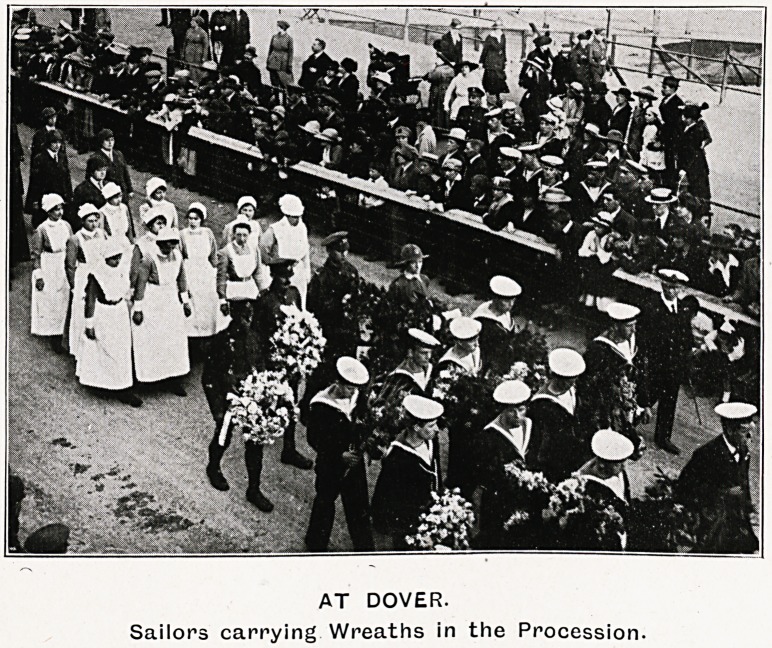


**Figure f4:**
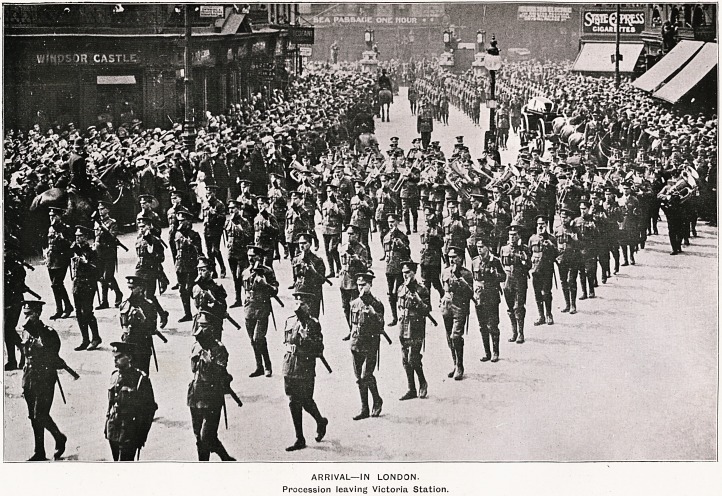


**Figure f5:**
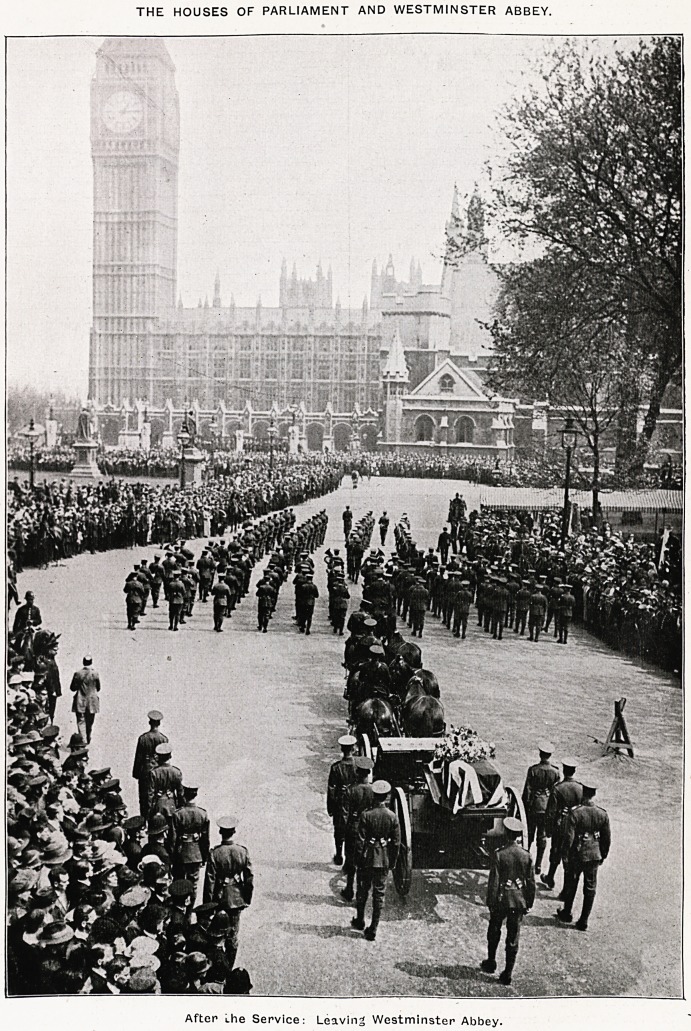


**Figure f6:**